# Effectiveness of multimodal active physiotherapy for chronic knee pain: a 12-month randomized controlled trial follow-up study

**DOI:** 10.3389/fphys.2024.1451345

**Published:** 2024-11-20

**Authors:** Xinwen Cui, Peng Zhao, Xuanhui Guo, Jialin Wang, Tianran Han, Xiaoya Zhang, Xiao Zhou, Qi Yan

**Affiliations:** ^1^ Sports Rehabilitation Research Center, China Institute of Sport Science, Beijing, China; ^2^ Graduate School, Beijing Sport University, Beijing, China; ^3^ Rehabilitation Center, Beijing Dynamic Tech Clinic, Beijing, China

**Keywords:** multimodal physiotherapy, active physiotherapy, knee pain, functional recovery, functional training

## Abstract

Active physiotherapy (APT) embraces a patient-centered approach, prioritizing self-management within the biopsychosocial model and involving active patient movements. Beyond structured exercise, APT incorporates pain neuroscience education, Mulligan Mobilization (MWM), and active myofascial release techniques to integrate sensory-motor information for functional recovery and pain relief. This study aims to rigorously compare the effectiveness of APT *versus* conventional physical therapy (CPT) on pain and functional outcomes in patients with chronic knee pain. Eighty-seven patients with symptomatic and radiographically confirmed knee pain were included in this 12-month follow-up of a randomized controlled trial, conducted at a national institute and a rehabilitation clinic. Patients were randomized to either APT (n = 44) or CPT (n = 43). The APT protocol integrated pain neuroscience education, MWM, active myofascial release techniques, and structured exercises focusing on flexibility, stability, neuromuscular control, and coordination. The CPT protocol included health education, laser therapy, ultrasound therapy, and exercise. Both interventions were performed for 60 min twice a week for 3 months. The primary outcome was the Knee Injury and Osteoarthritis Outcome Score-4 domain version (KOOS4). Secondary outcomes included pain intensity (VAS), KOOS-pain, activities of daily living (ADL), function in sport and recreation (Sports/Rec), knee-related quality of life, global rating of change (GROC), quality of life (SF-36), Tampa Scale for Kinesiophobia (TSK), and functional performances measured at different intervals. Intention-to-treat analyses were performed. Of the 87 patients, 70 (80.5%) completed the 12-month follow-up. KOOS4 improved more in the APT group (16.13; 95% CI, 10.39–21.88) than in the CPT group (11.23; 95% CI, 5.42–17.04). APT showed additional improvement in KOOS4 compared to CPT (2.94; 95% CI: 0.04 to 5.85, *p =* 0.047). The VAS difference was −3.41 mm (95% CI: −6.40 to −0.43, *p* = 0.025), favoring APT. APT also showed more improvements in KOOS-pain, KOOS-ADL, KOOS-Sports/Rec, and TSK (*p <* 0.05). No differences between groups were observed in GROC and SF-36. APT significantly improved most functional performance variables compared to CPT (*p <* 0.05). Active Physiotherapy outshines conventional physical therapy by delivering more substantial reductions in pain intensity and marked enhancements in function among patients with knee pain. This distinctive efficacy underscores the invaluable role of APT in the management of chronic knee pain. By actively involving patients in their recovery journey, APT not only fosters superior results but also emphasizes the critical need to integrate these advanced therapeutic strategies into everyday clinical practices.

## 1 Introduction

Knee pain is the second most commonly reported area of chronic musculoskeletal pain after back pain. Over the past 20 years, its incidence has increased by more than 65%, affecting over 25% of adults. This condition results in pain, balance problems, and functional limitations (Nguyen et al., 2011).

Leading institutions, including the Agency for Healthcare Research and Quality (AHRQ), the American College of Physicians (ACP), the Institute of Medicine (IOM), and the National Pain Strategy Report, recommend starting the treatment of chronic musculoskeletal pain with non-drug therapies and a biopsychosocial model, instead of depending exclusively on the conventional biomedical model. This strategy highlights self-management and biopsychosocial interventions (Hawk et al., 2020; Skelly et al., 2018).

Chronic knee pain is considered multifactorial and can be influenced by functional impairments (Melzack, 1999; Tsakiris, 2010; Lardner, 2010), physiological and psychological factors. Therefore, treatment strategies for knee pain should extend beyond a singular focus on the affected joint. While joint-targeted treatments are still appropriate, it is essential to properly manage other contributing factors (e.g., neuromuscular control, central mechanisms, psychosocial factors) that may influence an individual’s pain experience.

Pain is the primary symptom and a leading cause of disability. Based on present evidence-based clinical guidelines, contemporary nonpharmacological physical therapy for knee pain emphasizes alleviating symptoms through health education, exercise therapy, and, when necessary, weight management. Physical modalities (such as ultrasound, laser therapy, etc.) and manual therapy (including joint mobilization, myofascial release, etc.) act as supplementary treatments. (Zhu et al., 2023; Teo et al., 2019; Arden et al., 2021).

Exercise therapy is recommended as a core treatment. However, existing literature often emphasizes strength and flexibility training, with limited focus on neuromuscular or proprioceptive training. Neuromuscular and proprioceptive training is crucial for correcting dysfunctions in brain sensorimotor networks and addressing muscle imbalances. When such training is included, it is frequently implemented in a simplistic manner. Furthermore, existing exercise therapy programs frequently lack entertaining and interactive components, which can boost patient involvement and motivation by making the exercises more enjoyable and socially engaging. Moreover, exercise programs should be conducted progressively to ensure safety, meet the goals of each stage, and enhance overall effectiveness. (Kaya et al., 2019; Stensrud et al., 2012; Tanaka et al., 2016). Therefore, the existing exercise schedule needs to be optimized.

Pain neuroscience education is a psychological intervention method aimed at reconceptualizing pain. Although it has been supported to improve negative psychological characteristics (Cuenca-Martínez et al., 2023), the evidence in individuals with chronic knee pain is still limited and primarily focuses on those undergoing knee arthroplasty (Larsen et al., 2024; Louw et al., 2019) and those with knee osteoarthritis (Lluch et al., 2018; Rabiei et al., 2023).

According to the guidelines, physical modalities such as laser therapy and ultrasound therapy should be used as complementary methods. Several pieces of studies indicate their effectiveness in improving knee pain, function, and quality of life (Wu et al., 2022; Nazari et al., 2019). Some physical modalities have demonstrated their role in modifying disease progression, primarily through their effects on subchondral bone and gene modulation in early-stage knee osteoarthritis (Letizia Mauro et al., 2021). However, these treatments primarily target specific painful structures (hardware issues) and are entirely passive, relying on therapists and therapeutic devices. The management of neuromuscular control and dysfunction (software issues) is equally, if not more, important (Lardner, 2010).

Active physiotherapy (APT) adopts patient-centered approaches and prioritizes self-management within the biopsychosocial model, involving active neuromuscular movements by patients. In addition to exercise therapy, APT may also include Mulligan Mobilization (MWM) and active myofascial release techniques to integrate sensory-motor information for functional recovery and pain relief.

Currently, research comparing APT with CPT is relatively limited, especially in terms of long-term follow-up data. Additionally, there is a lack of evidence on the long-term effects of multimodal physical therapy for knee pain (Miller et al., 2017). Therefore, a multimodal APT approach is welcomed (Skou and Roos, 2019) and anticipated to be compared with the conventional method. Our study aimed to compare the effects of a multimodal progressed APT program (including pain neuroscience education, neuromuscular exercise, Mulligan mobilization, and myofascial release with a conventional program (comprising health education, exercise, laser therapy, and ultrasound therapy) on patients with chronic knee pain over a 12-month follow-up period. Our hypothesis is that the progressive multimodal APT program will provide greater benefits in terms of knee pain and function compared to the conventional program.

## 2 Methods

### 2.1 Study design and ethical approval

This was a two-center, randomized controlled trial (RCT) with a 1:1 randomization ratio involving participants with knee pain at a national institute and a rehabilitation clinic. This trial is reported according to the CONSORT 2010 checklist (Hopewell et al., 2008). Written informed consent was obtained from all patients prior to their participation. The study protocol was prospectively registered at www. chictr.org.cn (ChiCTR2200065627) and approved by the Ethics Committee of the China Institute of Sport Science (20220926), according to principles established for research on human subjects in the Declaration of Helsinki.

### 2.2 Randomization, blinding and sample size calculation

Participants were randomly allocated using the research randomizer program (http://www.randomizer.org/, accessed on 26 November 2022). Random blocks of varying sizes (2–6 per block) were employed, with stratification by baseline factors: age (30–49 and 50–70 years) and knee pain areas (anterior/posterior/medial/lateral/generalized knee pain). While assessors, investigators, statisticians, and participants remained blinded to grouping, physical therapists were unblinded. Experienced assessors managed equipment and measurement methods. Data unblinding occurred post-analysis for final result interpretation. All evaluations and exercise training were conducted individually. Based on preliminary experiment, PASS 15 Power Analysis and Sample Size Software (2017, NCSS, LLC. Kaysville, Utah, United States) was used to calculate the sample size with the following parameters: a two-tailed test, an expected difference of 5 points in KOOS4, a significance level (α) of 0.05, a power (1 - β) of 0.8, and an anticipated dropout rate of 20%. The required number of samples was 40.

### 2.3 Participants

We recruited participants aged 30–70 years through social networks (like WeChat, Weibo, partner hospitals and clinics), specifically targeting patients experiencing persistent knee pain (≥3/10) during weightbearing activities for more than 3 months. They needed to be exercise-capable and understand Chinese. An experienced physiotherapist confirmed eligibility based on history, examination, and MRI. Exclusion criteria: fractures, amputation, cancer, neuropathic pain, fibromyalgia, non-chronic knee pain (e.g., rheumatoid arthritis, gouty arthritis, septic arthritis), acute trauma, knee surgery within 2 years, severe osteoarthritis [Kellgren Lawrence (1957) score of 4], taking analgesics, abnormal sensory, pain pattern unrelated to movements or activities, pregnancy, insensitive to temperature, and contraindications to therapy. The most symptomatic knee at baseline was the study knee. Evaluations occurred at baseline, 3-month, 6-month and 12-month follow up. Online questionnaires were sent every 4 or 8 sessions (up to 24 sessions).

### 2.4 Interventions

Both APT and CPT programs comprised 24 sessions (twice weekly for 3 months, 1 h each). APT was conducted at a national institute, CPT at a rehabilitation clinic. Compliance was measured by completed sessions out of 24. Excellent: 24+ sessions (100%), Satisfactory: 17–23 (80%–100%), Poor: 16 or fewer (<80%). In per protocol analysis, ≤16 was non-compliant.

The APT program integrates pain neuroscience education, emphasizing the physiology of the nervous system, the differences between acute and chronic pain, analgesia theory, pain coping skills, and practical application of these skills. It also includes MWM (Wayne Hing, 2015), active myofascial release James and Zhang (2018) and structured exercises that progressively target flexibility, stability, neuromuscular control, and coordination. The CPT program includes health education covering the anatomy and biomechanics of the knee, the etiology and symptoms of knee pain, the importance of a healthy lifestyle and exercise. It also incorporates high-energy laser therapy, ultrasound therapy, and exercises focusing on flexibility, sustained isometric strengthening (including the quadriceps and proximal hip girdle muscles), and aerobic activities. The detail of both programs is shown in Supplementary Materials.

### 2.5 Outcome measurements

#### 2.5.1 Primary outcome

The primary outcome was the KOOS4, defined as the average score for four of the five knee injury and osteoarthritis outcome score (KOOS) subscale scores covering pain, other symptoms, function in sport and recreation, and knee related quality of life. KOOS is a reliable and responsive measure for assessing knee pain in adults, sensitive to changes in pain and knee-related symptoms. It includes 42 items scored on a Likert scale from 0 to 4. In this study, we calculated the KOOS4 score ranging from 0 (worst) to 100 (best). The recommended MCID is 10 points (Collins et al., 2016; Roos and Lohmander, 2003).

#### 2.5.2 Secondary outcomes

##### 2.5.2.1 Visual analog scale (VAS)

Knee pain was assessed using a 100 mm visual analog scale (VAS), where 0 represented no pain and 100 represented the worst imaginable pain. Participants rated their average pain over the last week. The minimum clinically important difference (MCID) was set at 20 mm based on previous studies (Collins et al., 1997; Tubach et al., 2005). We also determined the proportion of participants reporting a pain score below 10 mm, indicating a pain-free or nearly pain-free condition.

##### 2.5.2.2 KOOS subscales

Four KOOS subscales for pain, activities of daily living (ADL), function in sport and recreation, and knee-related quality of life were separately calculated and transformed to a scale from 0 (worst) to 100 (best).

##### 2.5.2.3 Global rating of change (GROC)

Global rating of change measured the participant’s subjective global change using a 15-point scale, ranging from “A very great deal worse” to “A very great deal better” (Jaeschke et al., 1989). For analysis, the scores were dichotomized as a success if the score was +5 or higher, corresponding to “Quite a bit better”, “A great deal better”, or “A very great deal better” (Lewis et al., 2020).

##### 2.5.2.4 Short-form health survey 36-item (SF-36)

We used the Short-Form health survey 36-item (SF-36), a widely used patient-reported outcome tool, to assess quality of life. It is a self-report measure of functional health and wellbeing. The questionnaire includes assessments of bodily pain (BP), general mental health (MH), limitations in usual role activities due to emotional problems (RE), limitations in usual role activities due to physical problems (RP), limitations in physical function (PF), limitations in social activities due to physical or emotional problems (SF), and vitality (V) (Zhang et al., 2012). We calculated the average score ranged from 0 to 100, with higher score indicating better outcome.

##### 2.5.2.5 Kinesiophobia

The Tampa Scale of Kinesiophobia (TSK) is a self-reported questionnaire that assesses fear of injury based on fear avoidance behavior and fear of activity. TSK has 17 components. Each scale runs from one (strongly disagree) to four (strongly agree). The responses are added together to get a total score, with higher values indicating greater pain related fear. The total score ranges between 17 and 68, with 17 indicating no kinesiophobia, 68 indicating severe kinesiophobia, and 37 indicating the presence of kinesiophobia (Wei et al., 2015; Rabiei et al., 2023; Theunissen et al., 2020).

##### 2.5.2.6 Joint position sensation (JPS)

Joint proprioception was assessed using the Baiobit sensor (BTS, Italy) by measuring the angle difference obtained from repeated reference angles (Selfe et al., 2006). The ICC for angle difference was 0.88 (95% CI = 0.84–0.92). All patients were positioned in a prone position with their hands at their sides. The patient’s knee was passively moved from 0° to reference points at 30°, 45°, and 60° of knee flexion, with each position held for 10–15 s. Patients were instructed to indicate when they felt the target angle was achieved by saying “OK”. The differences between the targeted angles and the angles actively reproduced by the patients were recorded (Selfe et al., 2006). The same procedure was repeated starting at 90° of knee flexion, with the patient’s knee passively extended to reference points at 15°, 30°, and 60° of knee flexion. Each reference point was repeated three times, and the average value was calculated. The proprioception measurement was recorded as the average of six reference angles. The measurements were taken by the same assessor.

##### 2.5.2.7 Balance test

Balance ability was assessed using the single-leg stance with eyes closed. Patients stood on their affected leg with arms crossed in front of their chest. Timing stopped when the supporting foot lifted off the ground or the opposite foot touched the ground. The duration of maintaining balance on the affected leg was recorded in seconds, and the proportion of time longer than 30 s was calculated. The measurements were taken by the same assessor.

##### 2.5.2.8 Functional performance outcomes

Lower extremity function was evaluated by some reliable and valid performance tests (Maldaner et al., 2022; Stensrud et al., 2014; Villadsen et al., 2012; Yuksel et al., 2017; McDonald et al., 2013) including the 30-second chair stand test, stair-climb test (SCT), 40 m fast-paced walk time and 6-minute walk test (6MWT). Proprioception test, balance test, and functional performance tests were conducted at baseline and the end of intervention (3-month follow-up).

### 2.6 Statistical analyses

All statistical tests were performed using SPSS (version 22.0, SPSS Inc., Chicago, IL, United States), with alpha levels of 0.05. Results are presented as means and standard deviations, with 95% confidence interval (CI), or numbers (percentage). Independent sample *t*-test or Mann-Whitney test and chi-squared test were used for descriptive statistics, two-way repeated measures ANOVA (group × time) with adjustment for sex, age, BMI and baseline VAS were used to examine the effect of APT vs. CPT. A chi-square test was conducted to compare the proportion of participants in each group.

Considering that some participants might drop out midway, all of the data were analyzed by using intention-to-treat analysis (including all randomized participants). Intention-to-treat was regarded as the primary analysis. Participants who withdrew from the intervention were contacted immediately to investigate their reasons for dropping out and were encouraged to continue the measurements to minimize the loss of follow-up data. If the participants failed to follow-up or withdrew from the group, their last observation results were carried forward to fill in the missing data for intention-to treat analysis.

## 3 Results

**FIGURE 1:**
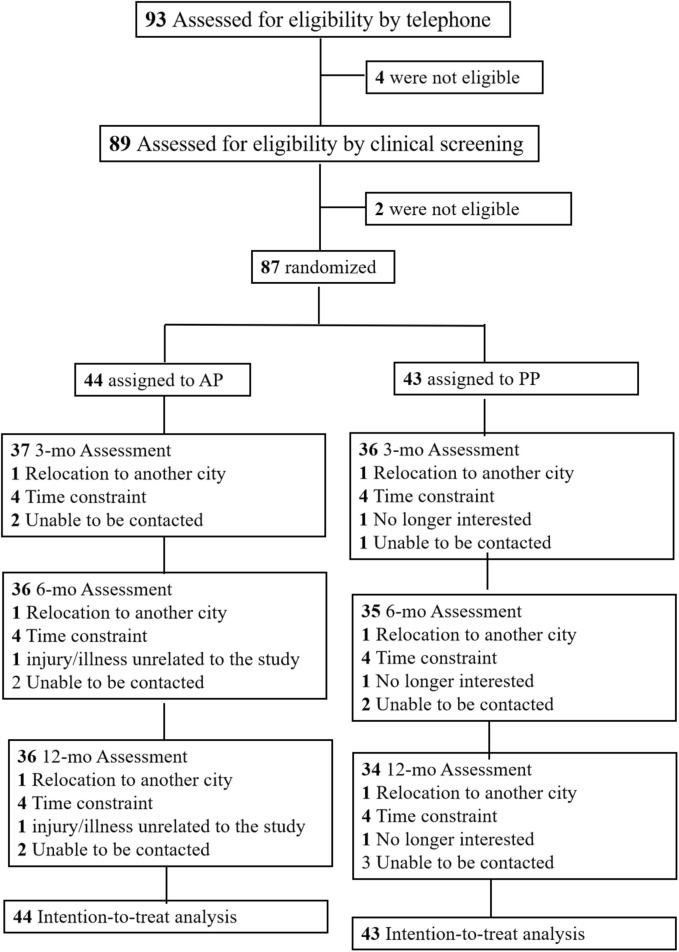
Flow chart of patients through the study.

This study occurred from 1 October 2022, to 16 October 2023. The trial flow is shown in Figure 1. Out of the 89 screened patients, 87 underwent randomization; 44 were assigned to the APT group and 43 to the CPT group. Of these, 70 patients (80.5%) completed the 12-month follow-up. In the APT group, 39 out of 44 participants (88.6%) completed the intervention program with satisfactory compliance, and 37 out of 44 participants (84.1%) achieved excellent compliance. Similarly, in the CPT group, 36 out of 43 participants (83.7%) completed the intervention program with satisfactory compliance, and 36 out of 43 participants (83.7%) achieved excellent compliance.

Loss to follow-up reasons were: relocation (n = 2); work/household commitments (n = 7); unrelated illness/injury (n = 1); caring for family (n = 1); no longer interested (n = 1) and unable to be contacted (n = 5). No side effects were reported during the12-month follow-up. Baseline traits (age, gender, BMI, pain areas, knee outcomes) were comparable. The mean (SD) age of the patients was 49 (11.3) years and 38 (43.7%) were men (Table 1).

**TABLE 1:** Baseline characteristics.

	AP (N = 44)		PP (N = 43)		
	Mean ± SD	Minimum- maximum	Mean ± SD	Minimum- maximum	*p*-value
**Demographics**					
Age (years), mean ± SD	48.11 ± 11.44	30–70	49.91 ± 11.15	31–70	0.377^a^
BMI (kg/m^2^), mean ± SD	23.49 ± 2.22	19.43–29.76	23.87 ± 2.87	18.20–30.09	0.442^a^
Gender (male), number (%)	19 (43.2%)	n/a	19 (44.2%)	n/a	0.925
Academic credentials					
High school equivalent (%)	6 (13.6%)	n/a	10 (23.3)	n/a	0.411
Bachelor’s degree (%)	21 (47.7%)	n/a	20 (46.6)	n/a	
Above bachelor’s degree (%)	17 (38.6%)	n/a	13 (30.2)	n/a	
**Knee pain areas**					
Anterior knee pain (%)	27 (61.4%)	n/a	26 (60.5%)	n/a	0.864
Medial knee pain (%)	5 (11.4%)	n/a	7 (16.3%)	n/a	
Lateral knee pain (%)	6 (13.6%)	n/a	4 (9.3%)	n/a	
Generalized knee pain (%)	6 (13.6%)	n/a	6 (14%)	n/a	
**Knee pain and function**					
VAS, mean ± SD	57.66 ± 15.60	29–91	56.35 ± 17.47	22–94	0.713
KOOS4, mean ± SD	57.92 ± 12.25	37.48–80.71	50.31 ± 14.16	21.89–76.88	0.626
SF-36, mean ± SD	70.33 ± 15.07	32.63–92.13	73.99 ± 16.63	25.13–93.75	0.220^a^
TSK, mean ± SD	44.75 ± 5.20	35–59	43.85 ± 5.33	33–56	0.422
30-Second Chair Stand Test (times)	15.07 ± 4.34	6–25	14.51 ± 4.69	7–24	0.397^a^
SCT (s), mean ± SD	12.07 ± 2.56	7.87–21.42	12.82 ± 3.43	7.83–22.99	0.255
6MWT (m), mean ± SD	559.48 ± 77.03	395–743	546.89 ± 80.25	352–758	0.457
40 m fast-paced walk time (s)	26.83 ± 4.92	19.97–41.41	26.83 ± 4.25	21.27–40.67	0.869^a^
Single-leg stance with eyes closed), mean ± SD	18.77 ± 13.51	3–50	15.65 ± 11.46	4–49	0.402^a^
JPS (°), mean ± SD	4.45 ± 1.76	1.67–9.5	4.50 ± 1.34	1.83–7.50	0.878

^a^ Mann-Whitney U test; AP, active physiotherapy; PP, passive physiotherapy; N = number of observations; BMI, body mass index; VAS, visual analog scale (range 0 (no pain) to 100 (worst pain imaginable); KOOS, knee injury and osteoarthritis outcome score; SCT, stair-climb test, 6MWT, 6-min walk test; JPS, joint position sensation.

### 3.1 Primary outcome

**FIGURE 2:**
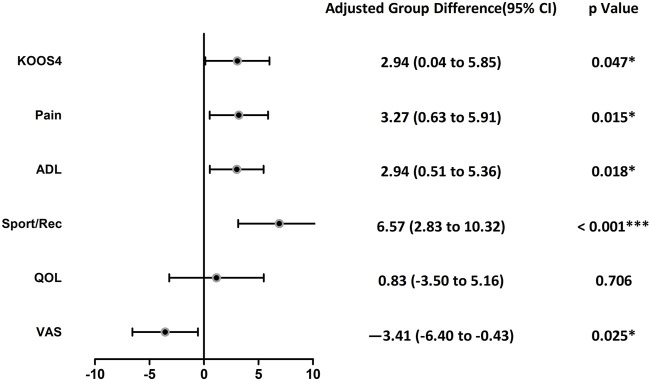
Forest plot of intention to treat analyses of differences between groups in KOOS subscales and VAS during the 12 months follow-up. KOOS4, Knee Injury and Osteoarthritis Outcome Score-4 domain version. VAS, visual analog scale, ADL, activities of daily living, Sports/Rec, function in sport and recreation, QOL, knee-related quality of life.

The KOOS4 scores increased and showed a significant (group effect (F = 3.963, *p =* 0.047) and time effect (F = 24.286, *p <* 0.001), but no interaction effect (*p =* 0.292). Compared with the patients in the CPT group, those in the APT group showed improvement by an additional 2.94 points (95% CI, 0.04 to 5.89, Figure 2; Table 2). The percentage of participants who met the MCID of a 10-point improvement for KOOS4 differed between the APT and CPT groups at the end of the 12-month follow-up (70.5% vs. 44.2%, χ^2^ = 6.140, *p <* 0.001).

**TABLE 2:** Comparisons of primary and secondary outcomes.

	AP (N = 44)		PP (N = 43)		Between-group Difference, adjusted means (95% CI)	*p*-value	η2
	Mean (SD)	Adjusted mean change (95% CI)^a^	Mean (SD)	Adjusted mean change (95% CI)			
**Primary outcome**							
KOOS4							
Baseline	57.92 ± 12.25		59.31 ± 14.15		−1.76 (−7.55 to 4.02)	0.549	
3-month	76.38 ± 13.91	18.46 (12.71–24.20)	70.91 ± 14.94	11.60 (5.79–17.41)	5.10 (−0.69–10.88)	0.084	
6-month	76.03 ± 13.12	18.11 (12.36–23.85)	70.36 ± 14.94	11.05 (5.24–16.87)	5.29 (−0.49–11.08)	0.073	
12-month	74.02 ± 12.43	16.13 (10.39–21.88)	70.51 ± 14.32	11.23 (5.42–17.04)	3.14 (−2.65–8.93)	0.287	
Overall	71.09 ± 14.90		67.77 ± 15.20		2.94 (0.04–5.85)	0.047^b^	0.012
**Secondary outcomes**							
Knee pain intensity (VAS, mm)							
Baseline	57.66 ± 15.60		56.59 ± 17.69		0.94 (−5.01–6.89)	0.633	
3-month	9.25 ± 9.56	−48.41 (−54.25 to −42.57)	15.57 ± 16.40	−40.62 (−46.96 to −34.93)	−6.52 (−12.44 to −0.60)	0.004^b^	
6-month	10.11 ± 10.74	−47.55 (−53.39 to −41.71)	13.40 ± 13.90	−42.79 (−49.12 to −37.10)	−349 (−9.41 to 2.45)	0.285	
12-month	13.36 ± 13.34	−44.31 (−50.17 to −38.50)	17.74 ± 15.19	−38.45 (−44.83 to −32.80)	−4.58 (-10.50 to 1.34)	0.16	
Overall	22.60 ± 18.85		25.64 ± 17.23		−3.41 (−6.40 to −0.43)	0.025^b^	0.017
KOOS-pain							
Baseline	72.47 ± 9.04		71.31 ± 13.84		0.94 (−4.32–6.4219)	0.726	
3-month	84.86 ± 11.56	12.39 (7.18–17.62)	79.93 ± 13.84	8.62 (3.34–13.89)	4.70 (−0.55–9.95)	0.079	
6-month	84.34 ± 11.01	11.87 (6.65–17.09)	79.52 ± 14.19	8.21 (2.94–13.29)	4.59 (−0.66–9.84)	0.087	
12-month	82.95 ± 11.20	10.48 (5.34–15.71)	79.87 ± 14.15	8.56 (3.35–13.90)	2.86 (−2.39–8.11)	0.285	
Overall	81.16 ± 11.80		77.66 ± 14.36		3.27 (0.63–5.91)	0.015^b^	0.019
KOOS- activities of daily living (ADL)							
Baseline	80.17 ± 9.75		80.42 ± 12.96		−0.92 (−4.74 to 3.91)	0.708	
3-month	89.73 ± 11.27	9.63 (4.84–14.42)	85.15 ± 12.90	4.73 (−0.11–8.59)	4.00 (−0.85–8.80)	0.106	
6-month	89.15 ± 9.23	8.99 (4.20–13.77)	83.16 ± 14.43	2.74 (−2.11–7.64)	5.30 (0.45–10.16)	0.032^b^	
12-month	87.45 ± 10.71	7.28 (2.72–12.30)	83.39 ± 14.11	3.20 (−1.65–8.04)	3.40 (−1.43–8.22)	0.167	
Overall	86.64 ± 10.89		83.03 ± 13.60		2.94 (0.51–5.36)	0.018^b^	0.019
KOOS-function in sport and recreation (Sports/Rec)							
Baseline	53.30 ± 16.98		51.05 ± 18.28		1.76 (−5.69–9.22)	0.642	
3-month	76.85 ± 16.19	23.55 (16.15–30.96)	67.44 ± 19.62	16.40 (8.91–23.88)	8.92 (1.46–16.39)	0.019^b^	
6-month	74.34 ± 15.55	21.05 (13.64–28.45)	63.84 ± 19.54	12.79 (5.30–20.28)	10.02 (2.56–17.48)	0.009^c^	
12-month	69.80 ± 16.69	16.50 (9.25–24.06)	63.72 ± 19.52	12.67 (5.33–20.31)	5.60 (−1.86–13.05)	0.14	
Overall	68.57 ± 18.65		61.51 ± 20.07		6.57 (2.83–10.32)	0.001^d^	0.03
KOOS- knee-related quality of life (QOL)							
Baseline	36.22 ± 22.25		43.45 ± 20.13		−7.58 (−16.20 to 1.04)	0.085	
3-month	65.69 ± 19.89	29.46 (20.90–38.02)	61.05 ± 21.30	17.59 (8.93–26.25)	4.29 (−4.33–12.91)	0.328	
6-month	67.05 ± 19.96	30.82 (22.26–39.38)	62.79 ± 20.99	19.34 (10.67–28.00)	3.91 (−4.71–12.53)	0.374	
12-month	68.04 ± 19.11	31.82 (23.14–40.27)	64.98 ± 20.53	21.52 (12.75–30.07)	2.72 (−5.91–11.35)	0.537	
Overall	59.25 ± 24.19		58.07 ± 22.28		0.83 (−3.50–5.16)	0.706	<0.001
Quality of life (SF-36)							
Baseline	70.17 ± 15.56		74.00 ± 16.63		−3,83 (−10.72 to 3.06)	0.275	
3-month	74.34 ± 14.34	4.17 (−2.60–10.94)	74.96 ± 17.89	0.96 (−6.05–7.97)	−0.63 (−7.51 to 6.26)	0.859	
6-month	74.85 ± 14.06	4.68 (−2.09–11.45)	74.81 ± 17.78	0.81 (−6.20–7.82)	0.03 (−6.86–6.92)	0.992	
12-month	75.70 ± 14.32	5.53 (−1.23–12.30)	72.55 ± 14.30	−1.45 (−8.5 to 5.60)	3.15 (−3.78–10.09)	0.372	
Overall	73.76 ± 14.61		74.09 ± 17.52		−1.18 (−4.41 to 2.06)	0.474	0.003
TSK							
Baseline	44.75 ± 5.20		44.00 ± 5.29		0.83 (−2.14–3.80)	0.541	
3-month	37.55 ± 7.50	−7.21 (−10.12to −4.30)	40.67 ± 7.81	−3.17 (−6.35 to −0.34)	−3.03 (−5.99 to −0.08)	0.044^b^	
6-month	37.91 ± 7.18	−6.84 (−9.75 to −3.93)	39.74 ± 7.10	−4.10 (−7.28 to −1.27)	−1.74 (−4.70 to 1.21)	0.223	
12-month	37.00 ± 7.18	−7.75 (−10.68 to −4.84)	40.07 ± 7.70	−3.76 (−6.96 to −0.95)	−3.07 (−5.94 to −0.028)	0.048^b^	
Overall	39.30 ± 7.47		41.10 ± 7.19		−1.73 (−3.22 to −0.25)	0.023^b^	0.017
30-Second Chair Stand Test (times)							
Baseline	15.07 ± 4.34		14.31 ± 4.56		0.53 (−1.51–2.57)	0.610	
3-month	19.89 ± 4.60	4.82 (2.81–6.83)	19.15 ± 6.37	4.80 (2.72–6.86)	0.55 (−1.50–2.60)	0.596	
Overall	17.48 ± 5.06		16.70 ± 8.01		0.54 (−0.91–1.99)	0.463	0.003
SCT (s)							
Baseline	12.08 ± 2.57		12.86 ± 3.50		−0.59 (−1.70 to 0.52)	0.296	
3-month	10.36 ± 2.24	−1.71 (−2.97 to −0.50)	11.95 ± 3.56	−0.89 (−2.17 to 0.38)	−1.47 (−2.53 to −0.29)	0.014^b^	
Overall	11.22 ± 2.55		12.41 ± 3.52		−1.00 (−1.79 to −0.21)	0.014^b^	0.037
40 m fast-paced walk time (s)							
Baseline	26.83 ± 4.92		26.83 ± 4.31		0.28 (−1.28–1.85)	0.721	
3-month	23.93 ± 3.03	−2.99 (−4.44 to −1.35)	25.34 ± 3.78	−1.49 (−3.03 to 1.40)	−1.17 (−2.74 to 0.41)	0.145	
Overall	25.38 ± 4.33		26.09 ± 4.10		−0.44 (−1.55 to 0.67)	0.434	0.004
6MWT (m)							
Baseline	559.48 ± 77.03		546.77 ± 81.20		7.60 (−22.25–37.45)	0.616	
3-month	607.25 ± 68.89	47.77 (15.68–79.87)	561.99 ± 79.54	14.86 (−17.80–47.52)	40.41 (10.38–70.44)	0.009^c^	
Overall	583.36 ± 76.52		554.29 ± 80.26		24.01 (2.81–45.21)	0.051	0.03
Single-leg stance with eyes closed (s)							
Baseline	18.77 ± 13.61		15.62 ± 11.60		2.11 (−4.11–8.33)	0.503	
3-month	43.80 ± 19.13	25.02 (18.07–31.97)	27.20 ± 20.68	11.48 (5.12–18.84)	15.66 (9.36–21.95)	<0.001^d^	
Overall	31.28 ± 20.72		21.27 ± 17.56		8.89 (4.45–13.31)	<0.001^d^	0.088
JPS (°)							
Baseline	4.45 ± 1.76		4.57 ± 1.29		−0.06 (−0.70 to 0.58)	0.842	0.03
3-month	1.90 ± 1.47	−2.55 (−3.20 to −1.91)	2.77 ± 1.43	−1.80 (−2.41 to −1.11)	−0.82 (−1.50 to −0.18)	0.012^c^	0.59
Overall	3.17 ± 2.06		3.67 ± 1.65		−0.44 (−0.89 to −0.01)	0.046^b^	0.023

AP, active physiotherapy; PP, passive physiotherapy; N, number of observations; 95% CI, 95% confidence interval, i.e., a 2-sided alpha of 5%; MICD, minimum important change difference; VAS, visual analog scale (range 0 (no pain) to 100 (worst pain imaginable); KOOS, knee injury and osteoarthritis outcome score; GROC, global rating of change; SF-36, short-form health survey 36-item; TSK, tampa scale for kinaesiophobia; SCT, stair-climb test; 6MWT, 6-min walk test; JPS, joint position sensation.

^a^ Adjusted for sex, age, BMI, and baseline VAS.

^b^ *p <* 0.05.

^c^ *p <* 0.01.

^d^ *p <* 0.001.

### 3.2 Secondary outcomes

Outcomes are presented in Table 2 and Figure 2. The knee pain intensity decreased and showed a significant group effect (F = 5.080, *p =* 0.025) and time effect (F = 212.356, *p <* 0.001), but no interaction effect (*p =* 0.350). Compared with the patients in the CPT group, those in the APT group showed improvement in pain intensity by an additional −3.41 mm (95% CI, −6.40 to −0.43). Additionally, the proportion of participants with a pain score below 10 mm, indicating minimal or no pain, was higher in the APT group compared to the CPT group at the 12-month follow-up (56.8% vs. 35.7%, *p* = 0.050).

Both APT and CPT groups showed significant changes over time in KOOS-pain (time effect, F = 14.283.514, *p <* 0.001), KOOS-activities of daily living (time effect, F = 6.669, *p =* < 0.001), KOOS-function in sport and recreation (time effect, F = 21.957, *p <* 0.001), KOOS-quality of life (time effect, F = 33.076, *p <* 0.001) and the Tampa Scale for Kinesiophobia (time effect, F = 13.739, *p* < 0.001). However, no significant time differences were found in SF-36 scores (*p* > 0.05).

Compared with the CPT group, those in the APT (group showed more improvements on the KOOS-pain (group effect, F = 5.953, *p =* 0.015), KOOS- activities of daily living (group effect, F = 5.680, *p =* 0.018), KOOS-function in sport and recreation (group effect, F = 11.919, *p =* 0.001) and TSK (group effect, F = 5.250, *p* = 0.023). Additionally, at the end of the 12-month follow-up, the percentage of participants without kinesiophobia was 54.5% in the APT group and 30.2% in the CPT group (χ^2^ = 5.259, *p* = 0.022). No significant group differences were found in KOOS-quality of life and SF-36 scores (*p* > 0.005).

Significant differences were observed in 30-second chair stand test (time effect, F = 43.246, *p <* 0.001), 40 m fast-paced walk time (time effect, F = 15.017, *p <* 0.001), stair-climbing (group effect, F = 6.228, *p =* 0.014; time effect, F = 10.707, *p =* 0.001), 6MWT (group effect, F = 4.997, *p =* 0.051; time effect, F = 8.583, *p =* 0.004), joint proprioception sense (group effect, F = 4.064, *p =* 0.046; time effect, F = 90.859, *p <* 0.001), and single-leg stance with eyes closed (group effect, F = 15.682, *p <* 0.001; time effect, F = 66.502, *p <* 0.001; overall group × time interaction effect, F = 9.157, *p =*0.003).

The proportion of participants reporting success on the GROC scale, indicating improvement [“Quite a bit better,” “A great deal better,” or “A very great deal better” (Lewis et al., 2020)], increased over time, with no difference between the groups (64.5% in the APT group *<*and 65.1% in the CPT group).

Adverse events were mild, with the most frequently reported being additional knee pain outpatient visits (5.6% in APT and 11.8% in CPT) and additional knee bruising outpatient visits (2.8% in APT and 11.8% in CPT).

## 4 Discussion

This randomized controlled trial demonstrated the superiority of progressively administered active physiotherapy compared to conventional physiotherapy in patients with chronic knee pain over a 12-month follow-up period. The study revealed significant differences between the groups in terms of knee pain and function.

In the context of supporting biopsychosocial, active, and self-management interventions, we established a multimodal active physiotherapy program combining pain neuroscience education, structured neuromuscular exercise, Mulligan mobilization, and myofascial release. We compared this active program with the conventional approach to assess their therapeutic effects on patients with knee pain. Both APT and CPT programs provided pain relief throughout the intervention period, likely due to shared pain reduction strategies.

The improvements in pain intensity and functional status can be attributed to the stimulation of non-nociceptive pathways, reduction of pain catastrophizing, and the release of endogenous analgesics (Ashar et al., 2022, David Butler, 2003; Lyman, 2021). Enhanced blood circulation, reduced inflammation and swelling, and improved range of motion also contributed to pain alleviation.

Notably, the APT group had a significantly higher proportion of pain-free participants, highlighting APT’s superiority in pain relief. Notably, the comparative improvement in pain between APT and CPT was significant, with a higher proportion of pain-free participants in the APT group, underscoring APT’s superiority in pain relief. In addition to the reasons mentioned above, APT may provide additional benefits that further reduce pain. These benefits could be related to the restoration of neuromuscular balance, correction of maladaptive pain cognition, behavior, movement patterns, and postures, reduction of joint and soft tissue stress, integration of sensory-motor information (Lardner, 2010), diffuse noxious inhibitory control (Bharadwaj et al., 2023; Samuelly-Leichtag et al., 2018), recovery of physical function, and correction of the excessive response to pain. This modulation of brain areas related to chronic pain and postural control might contribute to its effectiveness (Zhang et al., 2024). Ultimately, APT resulted in substantial pain relief.

In the APT program, we emphasized pain neuroscience education over general health education. Many individuals report confusion about the variability of their pain and its relationship with exercise, highlighting the need for strategies to address maladaptive beliefs and behaviors related to pain and physical activity. Pain neuroscience education is a cognitive-based and active physiotherapy intervention designed to enhance patients’ understanding of pain neurophysiology, reduce fear, and alter maladaptive thoughts and behaviors (Rice et al., 2019; Fechner et al., 2024). In this study, we incorporated pain neuroscience education at the start of the active physiotherapy program, resulting in decreased pain and improved TSK scores. These findings align with the known positive effects of pain neuroscience education on reducing catastrophizing and kinesiophobia in other chronic musculoskeletal pain populations (Larsen et al., 2024; Kasimis et al., 2024; Lendraitienė et al., 2024; Lluch et al., 2018; Sinatti et al., 2022). In this study, the Tampa Scale for Kynesiophobia (TSK) was used to assess kinesiophobia in knee pain patients. Although we believe that the current use of the TSK is justified based on its application in other knee osteoarthritis populations (Rabiei et al., 2023; Theunissen et al., 2020), we acknowledge the lack of validation of the TSK in Chinese knee pain patients. Future research should consider the validation of the TSK to ensure its cultural and contextual relevance.

Both groups in our current study showed improvements in physical function from baseline to 12-month follow-up, with the APT group demonstrating significantly better improvement, indicating the superiority of APT. The improved physical function in the CPT group might be attributed to the pain relief and physical exercise. While the APT program, designed for functional recovery training including the isometric and eccentric strength of muscles around the knee joint, stability control of hip external rotators and gluteus maximus, and stability control of core muscles such as transverse abdominis, internal and external obliques, pelvic floor muscles, diaphragm, and multifidus, lower limb movement pattern training and various neuromuscular training. These exercises build upon each other, systematically addressing weaknesses in the overall movement chain and aiding in the reestablishment of knee joint function through sensory-motor integration. As a result, better scores for subjective-reported function (KOOS scores) and most of the objective-based functional performances (SCT, 6MWT, JPS and balance ability) favored the administered APT protocol in our current study. Notably, the duration of single-leg stance with eyes closed in APT group was significantly longer early at the 3-month follow-up. These findings are consistent with other studies on therapeutic exercise (Holden et al., 2023; Torstensen et al., 2023; Kaya et al., 2019; Lu et al., 2021), suggesting that the superior outcomes may be attributed to symptomatic relief, improved muscle conditioning, and enhanced joint sense and balance achieved through functional training. Not only is this beneficial for the functional recovery of knee pain patients, but it could also play a crucial role in preventing future knee pain recurrence. Given that chronic pain management can benefit from lifestyle modifications (Sánchez Romero et al., 2022; Gonzalez-Alvarez et al., 2023), further studies are encouraged to examine the systemic effects of APT, including its impact on sleep quality and overall wellbeing.

Interestingly, despite the superior outcomes in the APT group compared to the CPT group, both groups of patients had similar self-evaluated effectiveness of change (GROC). This may be because patients in each group assessed their progress based on their individual pre-treatment condition, and both groups showed significant improvements compared to baseline during and after the treatment period. As a result, both groups of patients were highly satisfied with the APT or CPT interventions used in the study (77.1%, 76.5%, respectively).

Within the biopsychosocial framework and with an emphasis on self-management, our study illustrates that an active physiotherapy approach—integrating pain neuroscience education, patient-initiated neuromuscular exercises, and manual therapy to facilitate sensory-motor integration for functional recovery—is not only therapeutically effective but also cost-efficient. This method contrasts with traditional equipment- and therapist-dependent interventions. By replacing general health education with pain neuroscience education, incorporating patients’ active movements into manual therapy, and including structured exercises that focus on flexibility, stability, neuromuscular control, and coordination, we could propose a more holistic and efficacious strategy for managing chronic knee pain. Additionally, shifting from conventional to a more active physiotherapy model has potential for broader application in telerehabilitation. Furthermore, future research could extend these findings to other musculoskeletal conditions, thereby further substantiating the benefits of an active physiotherapy approach.

## 5 Limitations

The main limitation of this study is the absence of a negative control group not receiving any intervention, which would have allowed us to compare the results of both programs with the natural progression of knee pain. Additionally, the small to medium effect sizes observed in the current outcomes might be attributed to the relatively small sample size. Future studies should employ larger sample sizes to enhance the reliability of the findings.

It is also important to note that our participants were under 70, and for those over 70 years old, our APT program might be challenging. Moreover, the multimodal APT program in this study was restricted to pain neuroscience education, MWM, myofascial release, and therapeutic exercise. Future research should explore and validate other active physiotherapy approaches, such as dynamic neuromuscular stability techniques, postural restoration techniques, and cognitive behavioral therapy.

Besides, given the number of participants in this study, a team of four therapists was employed, with two therapists assigned to each group. All therapists underwent a rigorous 2-week training program prior to the study and successfully passed the subsequent evaluation. This model of standardized training can be replicated for future applications, thereby ensuring consistency and generalizability in wider clinical practice.

Additionally, this study did not investigate the underlying mechanisms of pain relief associated with active physiotherapy. Future research should delve into the mechanistic aspects to better understand how active physiotherapy exerts its analgesic effects.

## 6 Conclusion

Active physiotherapy demonstrates superiority in substantial pain relief, disability reduction, physical functional performance, balance ability, and joint position sensation improvement compared to conventional physiotherapy in patients with knee pain. Overall, our findings highlight the importance of activity in the treatment of chronic knee pain within the context of the biopsychosocial model. The active engagement of patients in their rehabilitation process is crucial for achieving better outcomes and underscores the value of incorporating active physiotherapy approaches in clinical practice. Furthermore, more studies are encouraged to examine the systemic effects of APT. Exploring its long-term advantages on wider health indicators will further underscore the importance of integrated, multifaceted approaches in managing chronic musculoskeletal conditions.

## Data Availability

The raw data supporting the conclusions of this article will be made available by the authors, without undue reservation.
