# Supplementary material for: Effectiveness of multimodal active physiotherapy for chronic knee pain: a 12-month randomized controlled trial follow-up study
Source: Front Physiol. 2024 Nov 20;15:1451345. doi: 10.3389/fphys.2024.1451345 (PMC11614761; doi:10.3389/fphys.2024.1451345)
Supplement: Supplementary file 1 [file Table1.docx]

Supplement 1. APT program in details

| **Session** | | **Objectives** | **Methods** | **Contents** | **Movement** | **Time** | |
| --- | --- | --- | --- | --- | --- | --- | --- |
| **Session 1-4** | | Alleviate pain and restore active range of motion in the lower extremities. | Pain neuroscience education MWM, myofascial release technique | **Pain neuroscience education (Session 1-2)**  Acute pain versus chronic pain, transition  from acute to chronic pain.  Neurons, synapses and action potentials  Peripheral and central sensitization.  Descending inhibition and facilitation of  Pain.  Sustaining factors of central sensitization  (i.e. emotions, stress, pain behavior,  cognitions).  The pain matrix in the brain.  Brain response to perceived threat.  Analgesia theory.  Aspects affecting nerve sensitivity.  Pain coping skills (progressive muscle relaxation, activity–rest cycling, pleasant activity scheduling, problem solving, identifying and challenging negative thoughts, developing coping thoughts, pleasant imagery, counting backwards, and auditory stimulation).  Transfer knowledge about pain to behavioral change.  **MWM**  Belt lateral glide MWM for knee flexion/extension, patellofemoral joint mobilization,  anteroposterior MWM for flexion.  Lateral distraction for hip flexion/extension/internal and external rotation.  Anteroposterior glide for ankle dorsiflexion, plantarflexion MWM. | With simultaneous knee or ankle flexion-extension, hip adduction-abduction. | Each session included 30 minutes of pain neuroscience education, and 20-50 minutes of MWM and myofascial release. The latter consisted of 2 sets of 1-minute interventions with a 30-second rest between each set. | |
|  |  |  |  | **Myofascial release technique**  Release of the quadriceps, hamstrings muscle group, adductor muscles, abductor muscles, iliopsoas, gluteal area，gastrocnemius, soleus and so on. |  |  |  |
| **Session 5-8** | | Restore active range of motion in the lower extremities (closed-chain exercises). | MWM (voodoo band)， myofascial release with myofascial ball or roller | **MWM**  Voodoo band lateral glide MWM for knee flexion/extension, anteroposterior MWM for knee flexion.  Voodoo band lateral distraction for hip flexion/extension.  Voodoo band anteroposterior glide for ankle dorsiflexion. | With simultaneous knee or ankle flexion-extension, hip adduction-abduction. | Each session consisted of 3 sets of 1-minute interventions with a 30-second rest between each set. |  |
|  |  |  |  | **Myofascial release**  Release of the quadriceps, hamstrings muscle group, adductor muscles, abductor muscles, iliopsoas, gluteal area，gastrocnemius, soleus and so on. |  |  |  |
| **Session 9-16** | | Restore stability control ability | Lower extremity isometric strength training, eccentric strength training, core stability training. | **Exercises**   - In the supine position   Hip bridge: Lie on back, knees bent, feet flat. Lift hips to form a straight line, then lower slowly.  Clamshell: Lie on side, knees bent, feet together. Lift top knee without moving pelvis, then lower slowly.  Side leg raise: Lie on side, legs straight. Lift top leg with resistance, then lower slowly with control.  Side plank: Lie on side, elbow under shoulder. Lift hips to form a straight line, hold as to be tired.   - In the sitting/kneeling position   Split squat horizontal push/pull: Start in a lunge, hold resistance band at chest. Push it forward, then pull back  Split squat overhead press: Start in a lunge, hold resistance band at chest. Push it diagonally overhead toward opposite shoulder, then lower slowly.  Bird-dog: Start on hands and knees. Gradually extend one arm, one leg and one hand with the opposite leg, then return.   - In the standing postion   Wall squat: Stand with back against wall with three 90°.  6-minute farmer's walk (with and without unstable surface), single-leg stance and crane balance.   - Movement patterns   Hip hinge: Stand with feet hip-width apart, hinge at hips, keep back straight, then return.  Sraight lunge: Step forward with one leg, lower body until both knees are at 90 degrees, then return.  Deep squat: Stand with feet shoulder-width apart, squat down as low as possible, keep back straight, then stand up  Single-leg calf raises: Stand on one leg, rise onto tiptoes, hold briefly, then lower slowly.  Crab walk: Sidewalks, keep the hips at the same level.  Relax with myofascial ball and foam roller. |  | Each session included 45 minutes dedicated to stability exercises and 15 minutes for foam rolling. Participants performed 2-3 sets of each exercise, with 10 repetitions per set. |  |
| **Session 17-20** | | Improving overall coordination | Bilateral unstable surface training with eye open or eye closed.  Unilateral unstable surface training with eye open or eye closed. | **Exercises**  Stepping/sitting-to-standing/squats/lunge squats on a Balance board.  Holistic coordination training system: stepping/walking for 6-8 minutes/double-leg squats/split-leg squats on the elastic bands.  Biodex training system for movement control training;  Relax with myofascial ball and foam roller. |  | Each exercise was performed for 1-2 sets, with each set lasting 1 minute.  Same as above |  |
| **Session 21-24** |  |  | **Exercises**  Single-leg standing/squat on a balance board.  Holistic coordination training system: single-leg hops/squats on the elastic bands.  Biodex training system：motor control ability training.  Relax with myofascial ball and foam roller. |  |  |  |  |

The APT program is conducted speciﬁc to patient’s needs. APT = active physiotherapy; MWM = Mulligan’s mobilization with movement.

Supplement 2. Conventional physiotherapy program in details

| **Session** | **Objectives** | **Methods** | **Contents** | **Time** |
| --- | --- | --- | --- | --- |
| **Session 1-8** | Alleviate pain and restore passive ROM of the knee joint. | Health education, ultrasound therapy/laser therapy, soft tissue release | **Health education (Session 1-2)**  Anatomy, physiology and biomechanics  of the knee.  Disease knowledge: normal course,  etiology and clinical presentation of knee  pain.  Principles of conservative management of knee pain: physical exercise, weight loss and healthy lifestyle.  **Ultrasound therapy**  Frequency: 1 MHz  Intensity: 1 W/cm² (watts per square centimeter)  Applicator diameter: 5 cm  Treatment duration: 5-10 minutes on each side of the knee  Patient position: Supine  Application method: Circular movements with the probe at right angles to ensure maximum energy absorption.  **Laser therapy**  Wavelength: 1064 nm  Power: 10W  Energy density of 10 J/cm2:  Total energy: 5000 J  Treatment duration: 10 minutes  Patient position: Supine with knee flexed at 30°  Application method: Circular movements with the probe placed vertically in contact with the joint line. | Health education for 20 minutes, laser therapy/ ultrasound therapy for 10 -15 minutes, soft tissue release for 10 minutes. |
|  |  |  | **Soft tissue release**  Release of the quadriceps, hamstrings muscle group, adductor muscles, abductor muscles. |  |
| **Session 9-24** | Alleviate pain and restore passive ROM of the lower extremities | Ultrasound therapy/laser therapy, soft tissue release, exercise | **Ultrasound therapy/ Laser therapy**  Same as above | Laser therapy/ ultrasound therapy for 10 -15 minutes, exercise for 30 minutes. |
|  |  |  | **Exercises**  **Flexibility**: stretch of quadriceps, hamstrings muscle group, adductor muscles, abductor muscles, iliopsoas, gluteal area，gastrocnemius, soleus.  **Strength**:  Wall Sits  Sit-to-Stand Exercises  Prone Leg Curls |  |
|  |  |  | **Activity (home-based)**: walking, aquatic exercise, running, cycling and so on. |  |

The conventional program is conducted speciﬁc to patient’s needs.
